# Right pulmonary artery occlusion by an acute dissecting aneurysm of the ascending aorta

**DOI:** 10.1186/1749-8090-1-29

**Published:** 2006-09-28

**Authors:** Ravi J De Silva, Reza Hosseinpour, Nicholas Screaton, Serban Stoica, Andrew T Goodwin

**Affiliations:** 1 Papworth Hospital NHS Trust, Papworth Everard, Cambridgeshire CB3 8RE, UK

## Abstract

We describe the case of a 76-year old female who presented with a Type A aortic dissection requiring repair with an interposition graft and aortic valve replacement. Post-operatively she had clinical features and computerised tomographic images suggestive of a pulmonary embolus and died 24 hours later. The extremely rare finding of intramural thrombus occluding the right pulmonary artery was seen at post mortem.

## Background

Acute type A aortic dissection is a serious and life-threatening condition that requires prompt diagnosis and surgical treatment. The presenting symptom of chest pain is often erroneously attributed to either myocardial infarction or pulmonary embolus. We describe an exceptionally rare presentation of acute type A aortic dissection combined with occlusion of the right pulmonary artery.

## Case report

**Figure 1:**
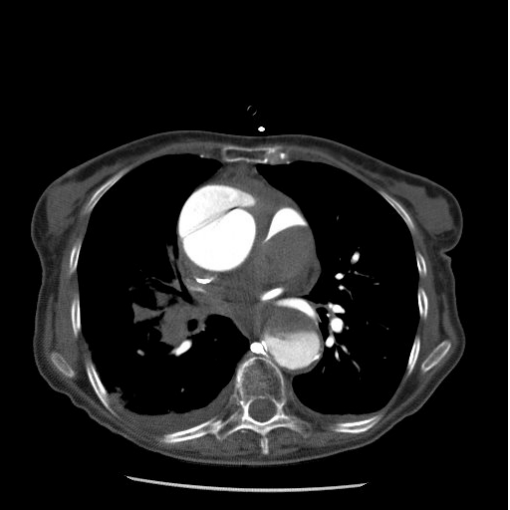
**CT image of occluded right pulmonary artery and aortic dissection**. Contrast enhanced CT image at the level of the right pulmonary artery demonstrates an aneurismal ascending aorta (AA) with a dissection flap and adjacent mediastinal haematoma. The right main pulmonary artery (RPA) is occluded.

A 76-year old woman with known hypertension developed sudden central chest pain radiating through to her back, and of a 'ripping' nature. She was referred to hospital with presumed ischaemic cardiac pain. On arrival in hospital she had a normal electrocardiograph and chest radiograph, however the pain was unremitting. With the exception of hypertension, physical examination was unremarkable. A transthoracic echocardiogram indicated the presence of a dissection flap in the ascending aorta, which was subsequently confirmed with contrast enhanced computerised tomography (CT) of the chest (figure 1). The full extent of the dissection was from the aortic valve to just beyond the left subclavian artery, and the peri-aortic haematoma appeared to be compressing the right pulmonary artery.

**Figure 2:**
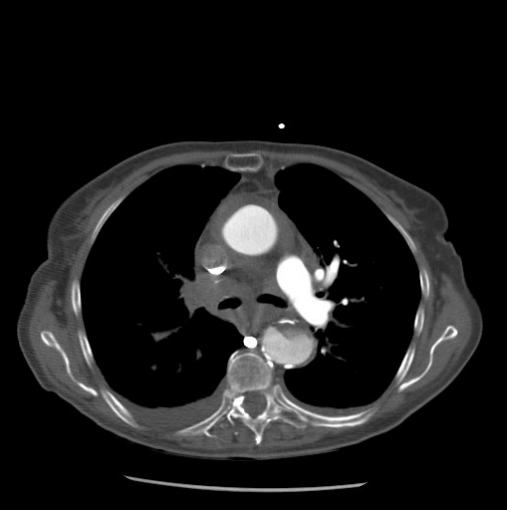
**CT image showing compression of the right pulmonary artery following repair of the aortic dissection**. This image demonstrates the aortic graft (G) and the peri-aortic haematoma still compressing the right pulmonary artery (RPA).

Following admission to our institution the patient was transferred to the operating theatre emergently and underwent aortic valve replacement (21 mm Perimount Magna, Edwards Lifesciences, CA, USA) and interposition graft replacement of the ascending aorta (30 mm Haemashield, Boston Scientific, MA, USA), reinforced with felt strips. This was done without circulatory arrest, with the aortic remnant wrapped around the graft. Weaning off bypass was difficult due to high venous and low systemic arterial filling pressures, and the main pulmonary artery (PA) was noticed to be very tense. A trans-esophageal echocardiogram (TEE) suggested the presence of thrombus within the main PA. The main PA was opened and revealed no intraluminal thrombus. However there was a mass compressing the PA posteriorly. This compression was overcome by widening the PA using a bovine pericardium patch (GlaxoSmithKline, Middlesex, UK), the subsequent appearance on TEE being much improved. The patient was successfully weaned off bypass and transferred to the intensive care unit. However, twelve hours post-operatively the patient became increasingly hypoxic with a respiratory acidosis, and haemodynamically unstable requiring increasing levels of inotropic support. These features were suggestive of an acute pulmonary embolus. A contrast enhanced CT scan of the chest showed an occlusion of the right PA (figure 2), similar to what was seen intra-operatively with TEE. However the poor clinical status of the patient precluded any consideration of surgical thrombectomy or stent placement within the PA. The patient eventually died 24 hours post surgery.

**Figure 3:**
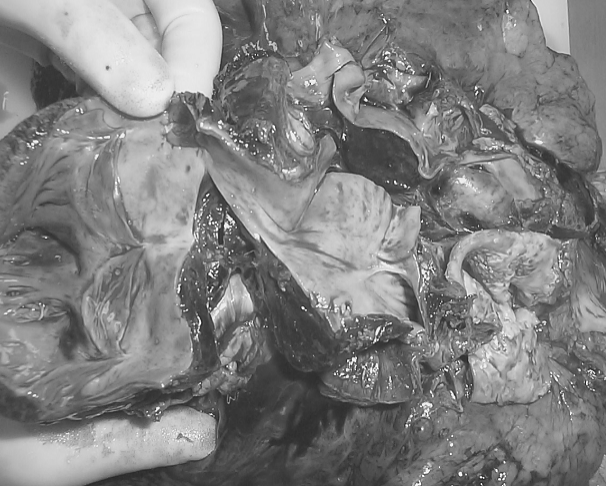
**Autopsy specimen showing thrombus compressing right pulmonary artery**. The autopsy specimen clearly shows the thrombus (T) extrinsically compressing right pulmonary artery (RPA). Also shown is the cavity of the right ventricle (RV).

The post mortem examination confirmed that the aortic valve, interposition graft, and PA patch were correctly placed. However, there was the extremely rare finding of a thrombus extending from the aortic dissection and compressing the right PA (figure 3). The thrombus was beneath the adventitial layer shared by the aorta and PA, but not within the lumen of these vessels.

## Conclusion

Acute aortic dissections and pulmonary emboli are often diagnosed post-mortem [1]. Both conditions present with similar symptoms, and can be rapidly fatal. Occlusion of the pulmonary artery by an acute aortic dissection is an extremely rare finding [1-8]. It was first described by Buja and colleagues [5], since when only a few cases have been reported in the literature. The right PA is susceptible to extrinsic compression by the haematoma of an aortic dissection due to its anatomical relationship to the aorta and as it shares a common tunica adventitia, thereby enabling a haematoma to track back from the aorta and up the PA. The previously reported cases are in patients who were initially thought to have had only a PE, or many years following aortic valve replacement. In only three instances have the patients survived. In these cases the thrombus was either evacuated [3,4] or the pulmonary trunk replaced with a prosthesis [2].

We present a case of acute aortic dissection, which initially underwent successful surgical correction. Despite attempts to surgically enlarge the main PA, the patient developed signs consistent with an acute PE several hours post-operatively. Subsequent investigation revealed further compression of the main and right PA, possible as a result of anticoagulation during cardiopulmonary bypass. The poor clinical condition of the patient at this point made further aggressive management futile. Post mortem findings revealed an extremely rare scenario that would not have been amenable to surgical embolectomy or thrombolysis. The rarity of this clinical presentation may be due to the poor antemortem diagnosis of both aortic dissection and pulmonary embolus. We suggests that patients who present with a massive unilateral perfusion defect, or hypoxic patients with an acute aortic dissection should be investigated with both CT and pulmonary arteriography, so that this unusual presentation is not overlooked and the chance of a surgical cure missed.
